# Incidence of Chronic Kidney Disease Following Acute Coronavirus Disease 2019 Based on South Carolina Statewide Data

**DOI:** 10.1007/s11606-023-08184-6

**Published:** 2023-04-12

**Authors:** Roy O. Mathew, Jiajia Zhang, Xueying Yang, Shujie Chen, Bankole Olatosi, Xiaoming Li

**Affiliations:** 1 Division of Nephrology, Department of Medicine, Loma Linda VA Health Care System, Loma Linda, CA 92357 USA; 2 Department of Medicine, Loma Linda University School of Medicine, Loma Linda, CA 92357 USA; 3 South Carolina SmartState Center for Healthcare Quality, Arnold School of Public Health, University of South Carolina, Columbia, SC 29208 USA; 4 Department of Epidemiology and Biostatistics, Arnold School of Public Health, University of South Carolina, Columbia, SC 29208 USA; 5 Department of Health Promotion, Education and Behavior, Arnold School of Public Health, University of South Carolina, Columbia, SC 29208 USA; 6 Department of Health Services Policy and Management, Arnold School of Public Health, University of South Carolina, Columbia, SC 29208 USA

**Keywords:** acute kidney injury, chronic kidney disease, coronavirus, COVID-19, outcomes

## Abstract

**Background:**

Coronavirus disease 2019 (COVID-19) was associated with severe acute illness including multiple organ failure. Acute kidney injury (AKI) was a common finding, often requiring dialysis support.

**Objective:**

Define the incidence of new clinically identified chronic kidney disease (CKD) among patients with COVID-19 and no pre-existing kidney disease.

**Design Participants:**

The South Carolina (SC) Department of Health and Environmental Control (DHEC) COVID-19 mandatory reporting registry of SC residents testing for COVID-19 between March 2020 and October 2021 was included.

**Design Main Measures:**

The primary outcome was a new incidence of a CKD diagnosis (N18.x) in those without a pre-existing diagnosis of CKD during the follow-up period of March 2020 to January 14, 2022. Patients were stratified by severity of illness (hospitalized or not, intensive care unit needed or not). The new incidence of CKD diagnosis was examined using logistic regression and cox proportional hazards analyses.

**Key Results:**

Among patients with COVID-19 (*N* = 683,958) without a pre-existing CKD diagnosis, 8322 (1.2 %) were found to have a new diagnosis of CKD. The strongest predictors for subsequent CKD diagnosis were age ≥ 60 years hazard ratio (HR) 31.5 (95% confidence interval [95%CI] 25.5–38.8), and intervening (between COVID-19 and CKD diagnoses) AKI diagnosis HR 20.7 (95%CI 19.7–21.7). The presence of AKI was associated with an HR of 23.6, 95% CI 22.3–25.0, among those not hospitalized, and HR of 6.2, 95% CI 5.7–6.8 among those hospitalized, for subsequent CKD. COVID-19 was not significantly associated with subsequent CKD after accounting for the severity of illness and comorbidities.

**Conclusion:**

Among SC residents, COVID-19 was not associated with CKD independent from indicators of the severity of illness, especially AKI diagnosis. Kidney-specific follow-up testing may be reserved for those high-risk for CKD development. Further prospective registries should examine the long-term kidney consequences to confirm these findings.

**Supplementary Information:**

The online version contains supplementary material available at 10.1007/s11606-023-08184-6.

## BACKGROUND

The novel 2019 coronavirus (CoV-2), responsible for the severe acute respiratory syndrome known as coronavirus disease 2019 (COVID-19), has caused a devastating 3-year-long pandemic. Of the ongoing challenges with COVID-19, many previously affected individuals reported sequelae long after the initial illness. Long COVID syndrome is a general phrase that is defined as symptoms associated with acute COVID-19 that persist for greater than 3 months.^1^ The most frequently reported symptoms include fatigue, dyspnea, cognitive and mental disorders, chest and joint pains, and gastrointestinal issues. These would be expected given the primary respiratory nature of the illness.

Despite initiating a primary respiratory infection, the ensuing illness was associated with multiple bystander organ effects including acute kidney injury (AKI). During the acute phase of COVID-19, AKI was seen in anywhere from 15 to 36% of patients; with the severity of the illness of COVID-19 correlating strongly with the incidence of AKI.^2,3^ Kidney pathologic evaluations in patients with COVID-19 and AKI have revealed acute tubular necrosis as the most common etiology. However, some cases have demonstrated direct viral invasion into kidney tissue.^4^ Thus, between the severe AKI and potential renal cell viral invasion, long-term kidney consequences remain a concern.

AKI is a known risk factor for incident CKD, and progression of established CKD.^5–7^ AKI occurring in the community or in the hospital is associated with a greater than 2–3-fold increased risk of progression to CKD or end-stage kidney disease (ESKD). Unfortunately, chronic kidney disease (CKD) is a silent illness and patients may not be aware that they have CKD.^8^ Given the prominence of AKI within the acute phase of the illness, a higher risk of subsequent CKD may be expected as part of this Long COVID syndrome. Whether the progression of CKD or development of new CKD would occur after COVID-19 independent of AKI or possibly direct pathologic viral entry into renal epithelial cells, is not clear.^9^ To address this issue and understand the relationship of COVID-19 to subsequent kidney dysfunction, the current analysis aims to examine the prevalence of new CKD diagnosis following COVID-19 using the South Carolina (SC) Statewide COVID-19 tested cohort. The hypothesis is that COVID-19 positivity would be associated with subsequent new CKD diagnoses independent of the severity of acute illness or development of AKI.

## METHODS

### Population and Data Source

The study population is SC residents who had ever tested for CoV-2 (both positive and negative) and reported to the South Carolina Department of Health and Environmental Control (SC DHEC) between March 2, 2020, and October 14, 2021 (i.e., COVID-19 tested cohort). SC DHEC uses SC statewide Case Report Form (CRF; “Human Infection With 2019 Novel Coronavirus Case Report Form”) to collect CoV-2 infection information^10^ which contains disease status (lab-confirmed case or probable case), hospitalization, intensive care units (ICU), and death information, case demographics, and symptoms (self-reported). Probable cases were defined according to strict DHEC criteria to include meeting clinical criteria and epidemiologic evidence, meets presumptive lab evidence (detection of a specific antigen in a clinical specimen, or detection of specific antibody in serum, plasma, or whole blood indicative of a new or recent infection), or meets vital records criteria with no confirmatory lab testing.

**Figure 1:**
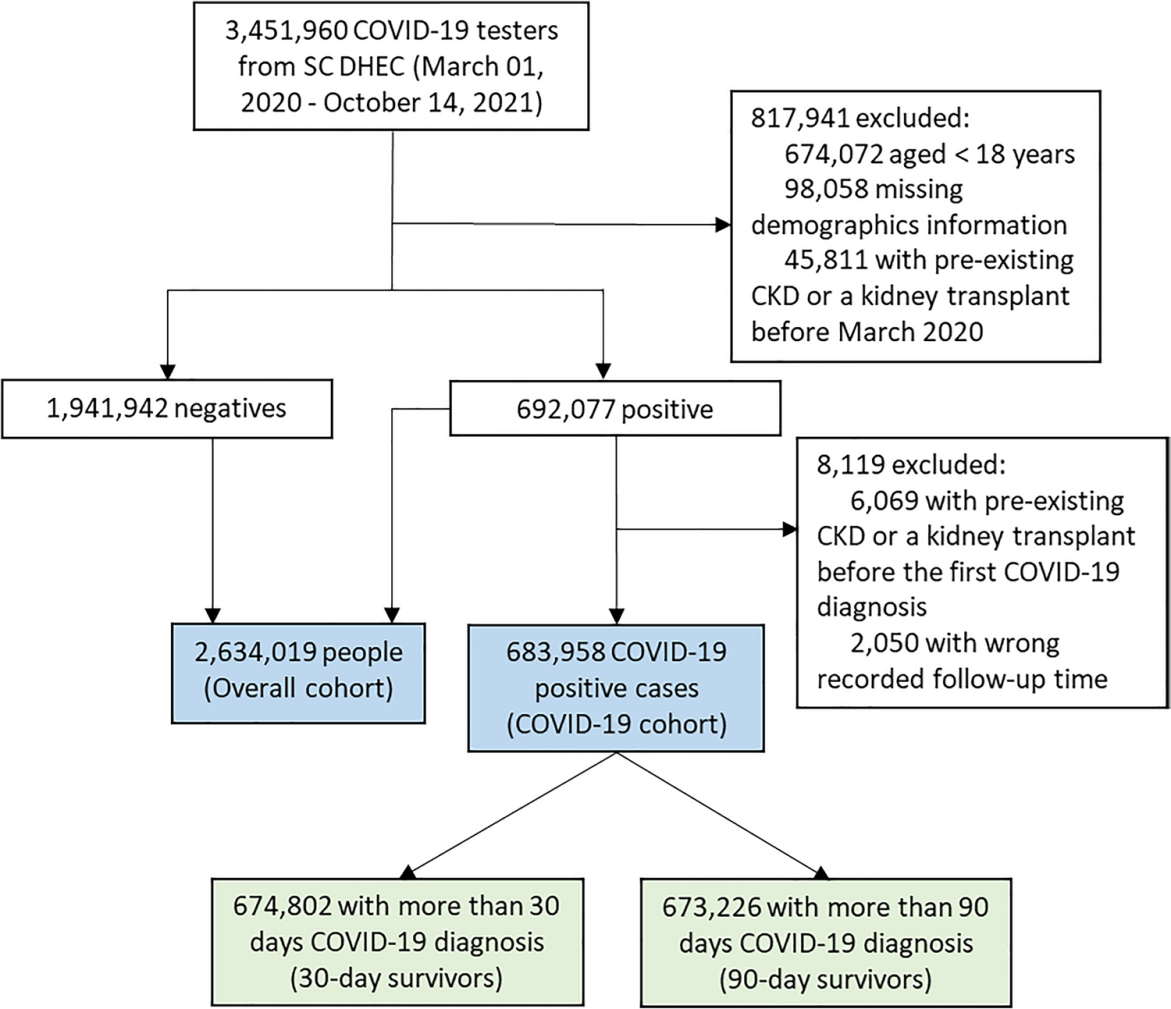
Flow chart for population inclusion criteria.

This cohort was integrated with multiple electronic health records (EHR) by the SC Office of Revenue and Fiscal Affairs (RFA), which is a state agency that receives reports on all diagnoses in the Uniform Billing form (UB-92) from all emergency departments, hospital inpatient facilities, ambulatory-care facilities, and outpatient surgery facilities in SC. To extract all the clinical diagnoses (e.g., CKD diagnosis) recorded in EHR prior to the COVID-19 outbreak, over one year of healthcare encounter information (January 1, 2019–January 14, 2022) was extracted. The look-back period was a minimum of 1 year between March 2019 and March 2020 for all patients. For those testing positive, a look back extending from January 2019 to the date of the test was used. Thus, there was an additional exclusion for CKD in the testing positive cohort (Fig. 1) if they had a diagnosis after March 2020 and there was evidence of a CKD diagnosis between March 2020 and their test positive date. The research protocol received approval from the institutional review board at the University of South Carolina and relevant SC state agencies.

### Overall Cohort Building and Measures

To examine the impact of the COVID-19 pandemic on the incidence of CKD, the overall cohort consists of all those tested for CoV-2, without a CKD diagnosis (N18.x) before the COVID-19 pandemic (March 2020 as a cutoff when the first COVID-19 case was detected in SC). There was a total of 3,451,960 individuals who underwent COVID-19 testing, and among them, there are 45,811 individuals with pre-existing CKD diagnosis or with a kidney transplant before the COVID-19 pandemic outbreak in SC. As shown in Figure 1, after excluding 674,072 individuals who were less than 18 years old, 98,058 with missing demographic information, and 45,811 with pre-existing CKD (see Measures below) or with a kidney transplant before the outbreak of COVID-19 in SC we have included 2,634,019 individuals (692,077positives and 1,941,942negatives) as the study population for the overall cohort analysis.

### Measures

The CKD diagnosis was defined as the presence of corresponding *International Classification of Diseases, 10*^*th*^* revisions* (ICD-10) diagnostic codes from all-payer claims database (Supplement Table S1). The incidence of CKD during the pandemic (March 01, 2020–January 14, 2022) was defined as any new CKD diagnosis during the pandemic.

Social demographics include age group (18–29, 30–39, 40–49, 50–59, 60+ years old), gender (female, male), race (White, Black, Asian), ethnicity (Hispanic/Latino, Non-Hispanic/Latino), residence (rural, urban). Rural residences are counties that are not designated as part of Metropolitan Areas by the Office of Management and Budget ^11^.

The potential clinical factor, acute kidney injury (AKI), was defined as the presence of the corresponding ICD-10 codes (Table S1) after the first COVID-19 diagnosis and before the outcome occurrence (i.e., CKD diagnosis). The end-stage kidney disease is defined by N18.6 and Z99.2 (ICD-10 code). Comorbidities like hypertension and diabetes before the pandemic were defined based on the ICD-10 codes (Table S1).

### CoV-2-Positive Cohort Building and Measures

To further investigate the predictors for CKD development among the CoV-2 positive population, we established a CoV-2 positive cohort population who were CKD-free before diagnosis and follow-up until the end of the study (January 14, 2022). In this CoV-2 positive cohort, we excluded individuals with a pre-existing CKD diagnosis (*n*=6069), and 2050 patients with wrong recorded follow-up time, eventually resulting in a total of 683,958 cases for CoV-2 positive cohort analysis.

### Measures

The primary outcome is time to CKD incidence, which is defined as the number of days from the first CoV-2 positive test to the first CKD diagnosis in the COVID-19-positive cohort. Similar social demographics were included. Comorbidities like hypertension and diabetes before COVID-19 diagnosis were defined based on the ICD-10 codes (see supplement Table S1).

COVID-19-related symptoms were grouped into three categories: asymptomatic, mild, moderate/severe. Patients with any one of three symptoms, difficulty breathing, developing pneumonia, or ARDS were grouped into Moderate/Severe. Individuals who had any other symptoms (e.g., fever, cough, chills, muscle aches, vomiting, sore throat, abdominal pain, loss of taste and smell, diarrhea, headache, fatigue) were in the Mild group. Patients who did not report any symptoms were categorized as Asymptomatic. There are three categories of responses to hospitalization, ICU, and respiratory support in CRF, “Yes,” “No,” or “Unknown.” We dichotomized all these three variables into “1” and “0”, “1” indicating a confirmed “Yes” response while “0” indicating a confirmed “No” or unknown status.

### Statistical Analysis

Descriptive statistics such as frequency and percentage were used to summarize the distribution of CKD across characteristics for the overall cohort and CoV-2 positive cohort. The differences in the outcomes between demographics subgroups were compared via the chi-square test or Fisher’s exact test when the chi-square test is not valid. The logistic regression is applied to investigate the association between CKD incidence and CoV-2 status adjusting for demographics among the overall cohort and odds ratio (OR) and 95% confidence interval (CI) were reported. The interaction between demographics and CoV-2 status was tested, and only the models with the statistically significant interaction term were presented. The Cox proportional hazards (PH) models were used to study the time to new CKD diagnosis for the CoV-2 positive cohort. The hazard ratio (HR) and 95% CI were calculated and presented through forest plots. The PH assumption was verified by plotting the logarithm of the cumulative hazard function versus follow-up time. To examine the risk factors of CKD beyond the acute phase of COVID-19 illness (long COVID), we created two subgroups by further excluding 9,156 patients who were diagnosed with CoV-2 for less than 30 days (30-day survivor subgroup, *n*=674,802) and 10,732 patients who were diagnosed less than 90 days (90-day survivor subgroup, *n*=673,226). Similar PH models were applied to these two subgroups. Additional sensitivity analysis was performed on those with only 1 documented infection (97.5% of our test-positive population). *P* values under 0.05 were considered statistically significant. All statistical analysis was conducted using statistical software, SAS version 9.4 (SAS Institute, Inc., Cary, NC, USA) and R 4.2.0 (R Core Team (2022). R: A language and environment for statistical computing. R Foundation for Statistical Computing, Vienna, Austria. URL https://www.R-project.org/.)

## RESULTS

### Overall Cohort

Of 2,634,019 CoV-2 tested in the overall cohort, 56,058 (2.1%) had a new CKD diagnosis during the COVID-19 pandemic (Table 1). Patients diagnosed with CKD during the COVID-19 pandemic were older (≥ 60 years old 80% vs only 26%, *P* < 0.0001), more likely to be male (53% vs. 45%, *P* < 0.0001), more likely to be Black (35% vs. 23%, *P* < 0.0001), and not of Hispanic or Latino ethnicity (75% vs. 56%,* P* < 0.0001), and more likely to live in a designated Rural county (19% vs. 14%, *P* < 0.0001) as compared to those who were not diagnosed with CKD during COVID-19 pandemic. There was no difference in CoV-2 positive test proportion in those with CKD and those without (26% positive cases among those who tested in both groups, *P* = 0.410).

**Table 1:** Demographic Characteristics of the Overall South Carolina Severe Acute Respiratory Syndrome Coronavirus 2 (CoV-2) Tested Cohort

Characteristics	Overall	No CKD	New CKD Diagnosed	*P* value
	*N*=2,634,019	*N* =2,577,961	*N* =56,058	
**Age group**				<.0001
18–29	631,832 (23.99)	631,292 (24.49)	540 (0.96)	
30–39	462,553 (17.56)	461,313 (17.89)	1240 (2.21)	
40–49	407,618 (15.48)	404,838 (15.70)	2780 (4.96)	
50–59	419,300 (15.92)	412,666 (16.01)	6634 (11.83)	
60+	712,716 (27.06)	667,852 (25.91)	44,864 (80.03)	
**Gender**				<.0001
Female	1,452,650 (55.15)	1,426,518 (55.34)	26,132 (46.62)	
Male	1,181,369 (44.85)	1,151,443 (44.66)	29,926 (53.38)	
**Race**				<.0001
White	1,475,058 (56.00)	1,439,735 (55.85)	35,323 (63.01)	
Black	600,806 (22.81)	580,928 (22.53)	19,878 (35.46)	
Asian	29,568 (1.12)	29,317 (1.14)	251 (0.45)	
Other/Unknown	528,587 (20.07)	527,981 (20.48)	606 (1.08)	
**Ethnicity**				<.0001
Not Hispanic or Latino	1,475,596 (56.02)	1,433,626 (55.61)	41,970 (74.87)	
Hispanic or Latino	104,655 (3.97)	103,809 (4.03)	846 (1.51)	
Unknown	785,942 (29.84)	776,632 (30.13)	9310 (16.61)	
Missing	267,826 (10.17)	263,894 (10.24)	3932 (7.01)	
**Residence**				<.0001
Rural	365,077 (13.86)	354,508 (13.75)	10,569 (18.85)	
Urban	2,268,942 (86.14)	2,223,453 (86.25)	45,489 (81.15)	
**CoV-2 status**				0.4097
Negative	1,941,942 (73.73)	1,900,528 (73.72)	41,414 (73.88)	
Positive	692,077 (26.27)	677,433 (26.28)	14,644 (26.12)	

Among the overall cohort, older age, male gender, and Black vs White race demonstrated greater odds of CKD development (Table 2). Asian race vs. White race, Hispanic or Latino vs. Not Hispanic or Latino, and urban residence demonstrated lower odds of CKD development. CoV-2 positivity alone was not associated with subsequent CKD diagnosis (OR 0.99, 95% CI 0.968–1.007; *P* = 0.199). Interaction between demographic features and CoV-2 test positive status was assessed. CoV-2 positivity was associated with greater odds of CKD development among those of Black race, and Hispanic ethnicity (Table 2). When CoV-2 positive status by Race/Ethnicity interaction was evaluated, CoV-2 positive status demonstrated a statistically significant OR 0.91 (95% CI 0.89–0.93) for race, and OR 0.98 (95% CI 0.96–0.997) for ethnicity.

**Table 2:** Multivariable Adjusted* Logistic Regression for CKD Among All CoV-2 Tested (Positive or Negative)

Characteristics	No interaction	Interaction with race	Interaction with ethnicity
	OR (95% C.I.)	OR (95% C.I.)	OR (95% C.I.)
**Age group**			
18–29	Ref.	Ref.	Ref.
30–39	2.967 (2.681, 3.282)	2.96 (2.676, 3.276)	2.966 (2.681, 3.282)
40–49	7.597 (6.927, 8.332)	7.59 (6.921, 8.324)	7.596 (6.927, 8.331)
50–59	17.398 (15.935, 18.996)	17.414 (15.949, 19.013)	17.399 (15.936, 18.997)
60+	72.643 (66.722, 79.089)	72.71 (66.783, 79.163)	72.663 (66.740, 79.110)
**Gender**			
Female	Ref.	Ref.	Ref.
Male	1.542 (1.515,1.568)	1.543 (1.517, 1.570)	1.541 (1.515, 1.568)
**Residence**			
Rural	Ref.	Ref.	Ref.
Urban	0.898 (0.878, 0.918)	0.898 (0.878, 0.918)	0.898 (0.878, 0.918)
**Race**			
White	Ref.	Ref.	Ref.
Black	1.821 (1.787, 1.854)	1.724 (1.687, 1.761)	1.821 (1.787, 1.854)
Asian	0.609 (0.537, 0.691)	0.596 (0.515, 0.689)	0.609 (0.537, 0.691)
Other/Unknown	0.106 (0.098, 0.115)	0.089 (0.081, 0.098)	0.106 (0.098, 0.115)
**Ethnicity**			
Not Hispanic or Latino	Ref.	Ref.	Ref.
Hispanic or Latino	0.838 (0.782, 0.899)	0.830 (0.773, 0.89)	0.783 (0.717, 0.856)
Unknown	0.505 (0.493, 0.517)	0.505 (0.493, 0.517)	0.500 (0.487, 0.513)
Missing	0.663 (0.641, 0.686)	0.664 (0.642, 0.687)	0.659 (0.636, 0.682)
**Covid status**			
Negative	Ref.	Ref.	Ref.
Positive	0.987 (0.968, 1.007)	0.910 (0.887, 0.933)	0.976 (0.955, 0.997)
Race*Covid status			
White		Ref.	NA
Black		1.228 (1.179, 1.279)	NA
Asian		1.096 (0.819, 1.466)	NA
Other/Unknown		2.287 (1.909, 2.739)	NA
Ethnicity*Covid status			
Not Hispanic or Latino		NA	Ref.
Hispanic or Latino		NA	1.210 (1.047, 1.397)
Unknown		NA	1.064 (0.998, 1.134)
Missing		NA	1.059 (0.933, 1.202)

*Adjusted for age, gender, race, ethnicity, and residence

### COVID-19 Positive Cohort

Among those testing positive for CoV-2 (*N* = 683,958), 8322 (1.2%) were identified as having a new CKD diagnosis during follow-up. When the population was stratified by minimum 30- and 90-day available follow-up, 0.56% (3808/674,802) and 0.41% (2749/670,477) had incident CKD diagnosis, respectively. The differences in baseline demographic features between those who developed CKD and those who did not were like those seen in the overall cohort (Table 3). Patients testing positive with subsequent CKD diagnosis were more likely to have Moderate/Severe symptoms at the time of testing (23.8% vs. 10.7%, *P* < 0.0001). Thirty-one percent of those with subsequent CKD were hospitalized for COVID-19, vs only 3% of those without subsequent CKD (P < 0.0001). Markers of the severity of illness (ICU utilization and requirement for ventilatory support) were also more frequent among those with later CKD (Table 3).

**Table 3:** Demographic and Clinical Characteristics Among CoV-2 Positive Cases

Characteristics	Overall	No CKD	CKD	*P* value
	*N* =683,958^*^	*N* =675,636	*N* =8322	
**Age group**				<.0001
18–29	176,626 (25.82)	176,535 (26.13)	91 (1.09)	
30–39	130,568 (19.09)	130,369 (19.30)	199 (2.39)	
40–49	117,483 (17.18)	117,006 (17.32)	477 (5.73)	
50–59	111,164 (16.25)	110,110 (16.30)	1054 (12.67)	
60+	148,117 (21.66)	141,616 (20.96)	6501 (78.12)	
**Gender**				<.0001
Female	376,331 (55.02)	372,496 (55.13)	3835 (46.08)	
Male	307,627 (44.98)	303,140 (44.87)	4487 (53.92)	
**Race**				<.0001
White	414,156 (60.55)	409,376 (60.59)	4780 (57.44)	
Black	169,297 (24.75)	165,893 (24.55)	3404 (40.90)	
Asian	7104 (1.04)	7068 (1.05)	36 (0.43)	
Other/Unknown	93,401 (13.66)	93,299 (13.81)	102 (1.23)	
**Ethnicity**				<.0001
Not Hispanic or Latino	492,632 (72.03)	485,375 (71.84)	7257 (87.20)	
Hispanic or Latino	40,927 (5.98)	40,744 (6.03)	183 (2.20)	
Unknown	125,377 (18.33)	124,651 (18.45)	726 (8.72)	
Missing	25,022 (3.66)	24,866 (3.68)	156 (1.87)	
**Residence**				<.0001
Rural	98,071 (14.34)	96,433 (14.27)	1638 (19.68)	
Urban	585,887 (85.66)	579,203 (85.73)	6684 (80.32)	
**Hypertension**				<.0001
No	588,729 (86.08)	583,999 (86.44)	4730 (56.84)	
Yes	95,229 (13.92)	91,637 (13.56)	3592 (43.16)	
**Diabetes**				<.0001
No	642,428 (93.93)	636,501 (94.21)	5927 (71.22)	
Yes	41,530 (6.07)	39,135 (5.79)	2395 (28.78)	
**AKI**				<.0001
No	665,986 (97.37)	662,434 (98.05)	3552 (42.68)	
Yes	17,972 (2.63)	13,202 (1.95)	4770 (57.32)	
**Symptom**				<.0001
Asymptomatic	399,921 (58.47)	395,383 (58.52)	4538 (54.53)	
Mild	209,543 (30.64)	207,738 (30.75)	1805 (21.69)	
Moderate/Severe	74,494 (10.89)	72,515 (10.73)	1979 (23.78)	
**Hospitalization**				<.0001
No	662,640 (96.88)	656,882 (97.22)	5758 (69.19)	
Yes	21,318 (3.12)	18,754 (2.78)	2564 (30.81)	
**ICU**				<.0001
No	680,300 (99.47)	672,557 (99.54)	7743 (93.04)	
Yes	3658 (0.53)	3079 (0.46)	579 (6.96)	
**Respiratory support**				<.0001
No	672,115 (98.27)	665,050 (98.43)	7065 (84.90)	
Yes	11,843 (1.73)	10,586 (1.57)	1257 (15.10)	
**Follow-up time (days) [Median (min, max)]**	356 (1, 680)	357 (1, 680)	23 (1, 622)	

*The overall cohort for analysis excluded those with pre-existing CKD (N = 6,069), and those without reliable follow-up time (N = 2,050)

**Figure 2:**
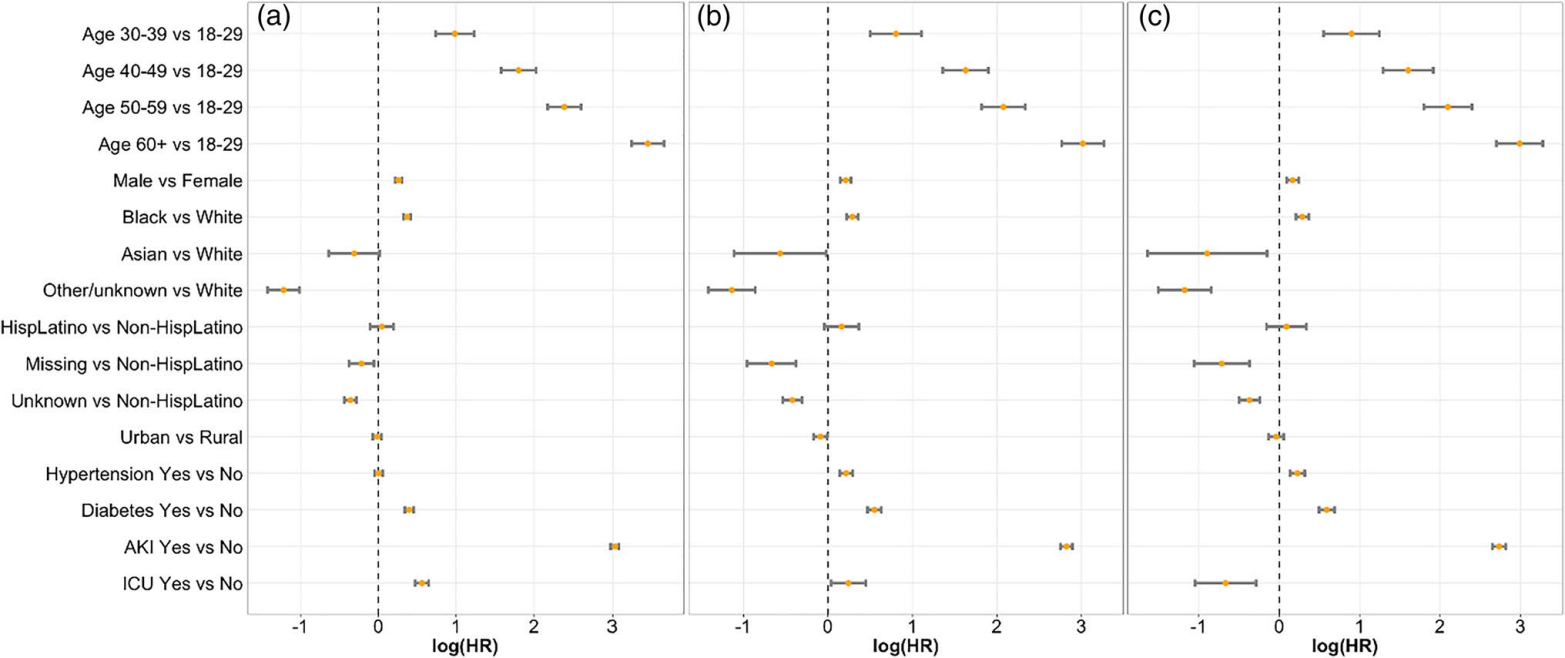
Forest plot for proportional hazard (PH) results for all, excluding people with less than 30 days follow-up, excluding people with less than 90 days follow-up. **a** Log(hazard ratio -HR) for chronic kidney disease (CKD) among all populations; **b** log(HR) for CKD when excluding follow-up time < 30 days; **c** log(HR) for CKD when excluding follow-up time < 90 days.

Among patients who were CoV-2 positive, older age (with 18–29 years old as reference) was associated with a graded increase (per 10-year interval) in risk for CKD development, with the highest risk for the ≥ 60 years old group (HR 31.5; 95% CI 25.6–38.8). Other significant positive risk factors for CKD included male gender, Black Race vs White, presence of diabetes, and requirement of ICU admission during hospitalization. The strongest predictor for subsequent CKD diagnosis after age ≥ 60 years, was intervening (between CoV-2 positive status and CKD diagnosis) AKI diagnosis with HR 20.7 (95% CI 19.7–21.7) (Fig. 2).

### Sensitivity Analysis

Given the potential for persistent AKI being mistaken for CKD, analyses were performed looking at individuals with > 30 days of follow-up and > 90 days of follow-up (current Kidney Disease: Improving Global Outcomes – KDIGO – guidelines for CKD diagnosis requires 3 months of abnormal kidney function tests to designate CKD status^12^) (Supplemental tables S2.1 and S2.2). The findings in these sensitivity analyses mirrored the main analysis; however, Asian race vs. White demonstrated a protective association for subsequent CKD (30-d: HR 0.57, 95% CI 0.33 – 0.98; 90-d: HR 0.41, 95% CI 0.19–0.86) (Fig. 2). Requirement of ICU stay demonstrated a protective effect for future CKD risk when > 90 days follow-up was required (Fig. 2c) HR 0.51 (95% CI 0.35–0.75).

Looking at those hospitalized (*N* = 21,318) versus non-hospitalized (*N* = 662,640), the associated HR was of similar directionality to the main analysis for those not hospitalized. However, AKI appeared to have a stronger effect on subsequent CKD for those not-hospitalized (HR 23.6, 95% CI 22.3–25.0) versus those who were hospitalized (HR 6.2, 95% CI 5.7–6.8) (Table S3) Finally, overall similar results were observed when restricted to those testing positive only once (Supplemental Tables S4.1 and S4.2).

### Development of End-Stage Kidney Disease (ICD 10 N18.6 or Z99.2)

A total of 452 patients were noted to have N18.6 and 279 with Z99.2 diagnostic codes following COVID-19 positivity. The demographics and proportion who died were similar to the overall population with non-dialysis CKD as the outcome (Supplemental Table S5.1 and S5.2).

## DISCUSSION

This is the largest cohort analysis examining the risk for the development of CKD following CoV-2 positive status to date. In the current analysis, we have demonstrated that the development of CKD over a median of 356 days following CoV-2 positive status was an uncommon event. Those who did develop CKD demonstrated standard risk factors for CKD development. Most importantly, there was not an association of CoV-2 positivity in the short term, independent of the development of AKI, with subsequent CKD diagnosis in those without a pre-existing CKD diagnosis. This has important implications for the case definitions for any long-term sequelae and resource utilization regards screening following CoV-2 infection

AKI being a frequent complication during the acute illness caused by CoV-2 infection, COVID-19, it would be expected that persistent renal dysfunction would be a feature during convalescence.^2,3^ AKI is known to predispose to the incident or progressive CKD.^13,14^ The mechanisms underlying the progression of AKI to CKD include tubulointerstitial scarring, the development of proteinuria, and the development of hypertension or worsening of blood pressure in hypertensives.^15,16^ CoV-2 infection with subsequent COVID-19 could predispose to CKD development through the AKI pathway, but there have been reports of direct viral invasion into the kidney suggesting that direct viral-induced cellular injury could independently cause remote kidney injury. The present analysis suggests that AKI in COVID-19, older age, Black race, and male gender, factors commonly reported as risk factors for CKD, and not CoV-2 positivity, were associated with future CKD.

Previously, several reports have suggested specific glomerular involvement, implicating direct viral injury to the kidney.^17,18^ The most striking example is the collapsing focal and segmental glomerulosclerosis seen in a subset of individuals.^18^ However, the presentation of these individuals was usually with some AKI and a marked increase in proteinuria. It was ultimately identified that the majority of patients with this presentation had high-risk APOL1 genotypes, thus predisposing them to glomerular injury in the setting of high inflammatory stress often seen with viral illnesses in general, not necessarily due to direct viral invasion.^18,19^ It remains to be proven, then, that CKD will be a long-term sequelae following COVID-19 independent of AKI, which was not the primary finding of the present analysis. Daugherty et al examined a large administrative health data set to examine new diagnoses following COVID-19.^20^ Among patients with COVID-19, as compared to historic controls, the risk of kidney injury (AKI or CKD) was higher among patients with COVID-19. Interestingly, they found in the stratified analysis, there was no increased risk for kidney injury in those not hospitalized as compared to those hospitalized, suggesting that the kidney injury outcome in their analysis was driven by the severity of illness, which is similar to the present analysis. Two separate, large, high-dimensional analyses in the same VA cohort, evaluated the risk of chronic conditions and CKD specifically, post-COVID-19.^21,22^ Al-Aly demonstrated that kidney conditions, primarily infections, were increased post-COVID-19; CKD was not noted as a significant outcome post-COVID-19; however, this analysis was not designed to explore kidney function outcomes specifically.^21^ Later, the same group examined the VA database and explored kidney function outcomes specifically.^22^ The outcomes of >30% - >50% decline in estimated glomerular filtration rate (eGFR), end-stage kidney disease, or death were more frequent among patients who were diagnosed with COVID-19 as compared to the rest of the VHA population. However, several stratification analyses identified that these risks were absent or attenuated for those not hospitalized, or hospitalized but without AKI, respectively.

The present analysis supports the hypothesis that after accounting for the severity of illness and the development of AKI, there does not appear to be an independent association between CoV-2 infection and the subsequent development of clinically evident CKD. The present analysis adds to the literature in several important ways. Despite the breadth of data available from the VHA system, the patient population is limited to typically older males, and primarily of either the White or Black race. The present analysis is from a large cohort in South Carolina, USA, with a similar racial background but with a wider range of ages and more gender parity. Furthermore, the present analysis was able to focus on new CKD diagnosis in those without a clinical diagnosis of pre-existing CKD. Lastly, using a contemporary control group that was naturally occurring (testing negative) allows for stronger temporal associations between the case and control groups. Thus, outside of acute illness, it is entirely possible that long-term effects in the kidney would be similar to a comparable cohort of uninfected individuals. This suggests that specific renal monitoring, outside of routine follow-up care with primary care providers, may not be necessary for all individuals post-COVID-19. Patients with AKI or requiring hospitalization during acute illness are likely at the highest risk for subsequent renal function changes and deserve close follow-up of renal function in the months following infection. In addition, older individuals may also be at higher risk for adverse changes to kidney function post-COVID-19.

On the other hand, it cannot be completely ruled out that lingering clinical sequelae following CoV-2 infection and specifically symptomatic COVID-19, may predispose to CKD. Daugherty and colleagues did find that the risk of subsequent hypertension and diabetes, known risk factors for CKD, was increased in individuals following CoV-2 infection, but more so among those hospitalized with COVID-19. Thus, further studies are needed to clarify the long-term risks of CKD. One such study of chronic illnesses post-COVID-19 is ongoing.( https://recovercovid.org/. Accessed 08232022 1041AM PST). Such studies may also provide insights into potential pathophysiologic mechanisms of kidney disease progression in infection if progressive kidney disease is indeed found.^23^ In addition, several baseline factors did associate with a higher risk for CKD including older age, male gender, and Black race versus White race, which is consistent with established epidemiologic data around CKD risk.^24^ Finally, the incidence of end-stage kidney disease was rare within the 12 months following COVID-19. In the present cohort, less than 1% were identified with this diagnosis during the available follow-up period. Longer follow-up would be needed to assess this outcome in more detail.

Several limitations need to be noted. Despite the large size of the cohort, specific parameters of renal function were not available for analysis, and diagnosis codes were relied upon to diagnose CKD. Incomplete case ascertainment or symptom ascertainment may be possible at the presentation. Incomplete ascertainment during follow-up may have led to an underassessment of the outcomes. This is important given the subtle nature of kidney injury that may be present (proteinuria) without an elevation in serum creatinine.^25^ The population represented in this analysis from South Carolina is predominantly White and Black (~76% of the entire cohort), thus limiting the generalizability to populations outside of this racial mix.

In conclusion, in a large, single US state cohort analysis of the post-COVID-19 incidence of CKD, there was no increased risk of clinically identified CKD among those with CoV-2 positivity as compared to those testing negative independent of the severity of the acute illness, or the concurrent development of AKI. Traditional risk factors such as older age, male gender, and Black race continued to be important independent predictors for subsequent CKD. There was evidence of interaction between CoV-2 positivity and these risk factors suggesting targeted follow-up of renal function in these high-risk groups post-CoV-2 infection may be warranted. Longer term studies with direct assessment of kidney function are necessary to verify the findings from this and other analyses.

## Supplementary Information

Below is the link to the electronic supplementary material.

Supplementary file1 (DOCX 42 KB)

## Data Availability

The University of South Carolina is prohibited from making individual-level data available publicly due to provisions in our data use agreements with state agencies/data providers, institutional policy, and ethical requirements. To facilitate research, we make access to such data available via approved data access requests. The data is unavailable externally or for re-release due to prohibitions in data use agreements with the South Carolina Department of Health and Environmental Control (SC DHEC). For more information or guidance on how to make a request, please contact (Bankole Olatosi, PhD):Olatosi@mailbox.sc.edu. The underlying analytical codes are available from the authors on request.
