# Supplementary material for: Incidence of Chronic Kidney Disease Following Acute Coronavirus Disease 2019 Based on South Carolina Statewide Data
Source: J Gen Intern Med. 2023 Apr 12;38(8):1911–9. doi: 10.1007/s11606-023-08184-6 (PMC10097447; doi:10.1007/s11606-023-08184-6)
Supplement: Supplementary file 1 — Supplementary file1 (DOCX 42 KB) [file 11606_2023_8184_MOESM1_ESM.docx]

Appendix:

Table S1: ICD-10 codes used for defining outcomes and comorbidities

| **Variable** | **Class** | **ICD-10 Diagnosis Code** |
| --- | --- | --- |
| CKD | Renal (Mild or Moderate) | I12.9, I13.0, I13.10, N03.x, N05.x, N18.1, N18.2, N18.3, N18.4, N18.9 |
|  | Renal (Severe) | I12.0, I13.11, I13.2, N18.5, N18.6, N19.x, N25.0, Z49.x, Z99.2 |
| AKI | Acute kidney injury | N17.x |
| Kidney transplant |  | Z94.0 |
| Hypertension |  | I10.x |
| Diabetes |  | E10.x, E11.x |

Table S2.1: Demographics and clinical characteristics when excluding people with less than 30-days follow-up.

| **Characteristics** | **Overall** | **No CKD** | **CKD** | **p-value** |
| --- | --- | --- | --- | --- |
|  | **N=674,802** | **N=670,994** | **N=3,808** |  |
| **Age group** |  |  |  | <.0001 |
| 18-29 | 176,559 (26.16) | 176,494 (26.30) | 65 (1.71) |  |
| 30-39 | 130,398 (19.32) | 130,282 (19.42) | 116 (3.05) |  |
| 40-49 | 117,061 (17.35) | 116,779 (17.40) | 282 (7.41) |  |
| 50-59 | 110,128 (16.32) | 109,582 (16.33) | 546 (14.34) |  |
| 60+ | 140,656 (20.84) | 137,857 (20.55) | 2,799 (73.50) |  |
| **Gender** |  |  |  | <.0001 |
| Female | 372,226 (55.16) | 370,355 (55.19) | 1,871 (49.13) |  |
| Male | 302,576 (44.84) | 300,639 (44.81) | 1,937 (50.87) |  |
| **Race** |  |  |  | <.0001 |
| White | 408,380 (60.52) | 406,163 (60.53) | 2,217 (58.22) |  |
| Black | 166,191 (24.63) | 164,669 (24.54) | 1,522 (39.97) |  |
| Asian | 7,052 (1.05) | 7,039 (1.05) | 13 (0.34) |  |
| Other/Unknown | 93,179 (13.81) | 93,123 (13.88) | 56 (1.47) |  |
| **Ethnicity** |  |  |  | <.0001 |
| Not Hispanic or Latino | 484,920 (71.86) | 481,601 (71.77) | 3,319 (87.16) |  |
| Hispanic or Latino | 40,670 (6.03) | 40,567 (6.05) | 103 (2.70) |  |
| Unknown | 124,470 (18.45) | 124,131 (18.50) | 339 (8.90) |  |
| Missing | 24,742 (3.67) | 24,695 (3.68) | 47 (1.23) |  |
| **Residence** |  |  |  | <.0001 |
| Rural | 96,306 (14.27) | 95,519 (14.24) | 787 (20.67) |  |
| Urban | 578,496 (85.73) | 575,475 (85.76) | 3,021 (79.33) |  |
| **Hypertension** |  |  |  | <.0001 |
| No | 583,355 (86.45) | 581,337 (86.64) | 2,018 (52.99) |  |
| Yes | 91,447 (13.55) | 89,657 (13.36) | 1,790 (47.01) |  |
| **Diabetes** |  |  |  | <.0001 |
| No | 635,537 (94.18) | 632,938 (94.33) | 2,599 (68.25) |  |
| Yes | 39,265 (5.82) | 38,056 (5.67) | 1,209 (31.75) |  |
| **AKI** |  |  |  | <.0001 |
| No | 661,969 (98.10) | 660,008 (98.36) | 1,961 (51.50) |  |
| Yes | 12,833 (1.90) | 10,986 (1.64) | 1,847 (48.50) |  |
| **Symptom** |  |  |  | <.0001 |
| Asymptomatic | 395,725 (58.64) | 393,561 (58.65) | 2,164 (56.83) |  |
| Mild | 207,985 (30.82) | 207,014 (30.85) | 971 (25.50) |  |
| Moderate/Severe | 71,092 (10.54) | 70,419 (10.49) | 673 (17.67) |  |
| **Hospitalization** |  |  |  | <.0001 |
| No | 658,660 (97.61) | 655,382 (97.67) | 3,278 (86.08) |  |
| Yes | 16,142 (2.39) | 15,612 (2.33) | 530 (13.92) |  |
| **ICU** |  |  |  | <.0001 |
| No | 673,176 (99.76) | 669,463 (99.77) | 3,713 (97.51) |  |
| Yes | 1,626 (0.24) | 1,531 (0.23) | 95 (2.49) |  |
| **Respiratory support** |  |  |  | <.0001 |
| No | 666,130 (98.71) | 662,554 (98.74) | 3,576 (93.91) |  |
| Yes | 8,672 (1.29) | 8,440 (1.26) | 232 (6.09) |  |
| **Follow-up time (days) [Median, min, max)** | 357, 31, 680 | 358, 31, 680 | 177, 31, 622 |  |

Table S2.2: Demographics and clinical characteristics when excluding people with less than 90-days follow-up.

| **Characteristics** | **Overall** | **No CKD** | **CKD** | **p-value** |
| --- | --- | --- | --- | --- |
|  | **N=673,226** | **N=670,477** | **N=2,749** |  |
| **Age group** |  |  |  | <.0001 |
| 18-29 | 176,542 (26.22) | 176,493 (26.32) | 49 (1.78) |  |
| 30-39 | 130,368 (19.36) | 130,274 (19.43) | 94 (3.42) |  |
| 40-49 | 116,951 (17.37) | 116,749 (17.41) | 202 (7.35) |  |
| 50-59 | 109,927 (16.33) | 109,515 (16.33) | 412 (14.99) |  |
| 60+ | 139,438 (20.71) | 137,446 (20.50) | 1,992 (72.46) |  |
| **Gender** |  |  |  | <.0001 |
| Female | 371,495 (55.18) | 370,105 (55.20) | 1,390 (50.56) |  |
| Male | 301,731 (44.82) | 300,372 (44.80) | 1,359 (49.44) |  |
| **Race** |  |  |  | <.0001 |
| White | 407,415 (60.52) | 405,824 (60.53) | 1,591 (57.88) |  |
| Black | 165,633 (24.60) | 164,522 (24.54) | 1,111 (40.41) |  |
| Asian | 7,037 (1.05) | * | * |  |
| Other/Unknown | 93,141 (13.84) | * | * |  |
| **Ethnicity** |  |  |  | <.0001 |
| Not Hispanic or Latino | 483,550 (71.83) | 481,169 (71.77) | 2,381 (86.61) |  |
| Hispanic or Latino | 40,617 (6.03) | 40,547 (6.05) | 70 (2.55) |  |
| Unknown | 124,343 (18.47) | 124,078 (18.51) | 265 (9.64) |  |
| Missing | 24,716 (3.67) | 24,683 (3.68) | 33 (1.20) |  |
| **Residence** |  |  |  | <.0001 |
| Rural | 95,964 (14.25) | 95,417 (14.23) | 547 (19.90) |  |
| Urban | 577,262 (85.75) | 575,060 (85.77) | 2,202 (80.10) |  |
| **Hypertension** |  |  |  | <.0001 |
| No | 582,492 (86.52) | 581,029 (86.66) | 1,463 (53.22) |  |
| Yes | 90,734 (13.48) | 89,448 (13.34) | 1,286 (46.78) |  |
| **Diabetes** |  |  |  | <.0001 |
| No | 634,396 (94.23) | 632,526 (94.34) | 1,870 (68.02) |  |
| Yes | 38,830 (5.77) | 37,951 (5.66) | 879 (31.98) |  |
| **AKI** |  |  |  | <.0001 |
| No | 661,206 (98.21) | 659,711 (98.39) | 1,495 (54.38) |  |
| Yes | 12,020 (1.79) | 10,766 (1.61) | 1,254 (45.62) |  |
| **Symptom** |  |  |  | <.0001 |
| Asymptomatic | 394,913 (58.66) | 393,374 (58.67) | 1,539 (55.98) |  |
| Mild | 207,665 (30.85) | 206,904 (30.86) | 761 (27.68) |  |
| Moderate/Severe | 70,648 (10.49) | 70,199 (10.47) | 449 (16.33) |  |
| **Hospitalization** |  |  |  | <.0001 |
| No | 657,627 (97.68) | 655,162 (97.72) | 2,465 (89.67) |  |
| Yes | 15,599 (2.32) | 15,315 (2.28) | 284 (10.33) |  |
| **ICU** |  |  |  | <.0001 |
| No | 671,804 (99.79) | 669,082 (99.79) | 2,722 (99.02) |  |
| Yes | 1,422 (0.21) | 1,395 (0.21) | 27 (0.98) |  |
| **Respiratory support** |  |  |  | <.0001 |
| No | 664,879 (98.76) | 662,241 (98.77) | 2,638 (95.96) |  |
| Yes | 8,347 (1.24) | 8,236 (1.23) | 111 (4.04) |  |
| **Follow-up time (days) [Median, min, max)** | 357, 91, 680 | 358, 91, 680 | 240, 91, 622 |  |

Note: * small number less than 10 were masked due to DHEC’s policy.

Table S3: Proportional Hazards results for new CKD diagnosis in SARS CoV-2 positive patients who were not-hospitalized and those who were hospitalized.

| **Characteristics** | **Not-hospitalized** | | **Hospitalized** | |
| --- | --- | --- | --- | --- |
|  | **HR (95% C.I.)** | **p-value** | **HR (95% C.I.)** | **p-value** |
| **Age group** |  |  |  |  |
| 18-29 | Ref. |  | Ref. |  |
| 30-39 | 2.511 (1.931, 3.267) | <.0001 | 2.081 (0.957, 4.528) | 0.0646 |
| 40-49 | 5.542 (4.370, 7.029) | <.0001 | 3.330 (1.617, 6.859) | 0.0011 |
| 50-59 | 9.896 (7.888, 12.416) | <.0001 | 4.563 (2.254, 9.238) | <.0001 |
| 60+ | 27.408 (21.998, 34.150) | <.0001 | 10.516 (5.244, 21.087) | <.0001 |
| **Gender** |  |  |  |  |
| Female | Ref. |  | Ref. |  |
| Male | 1.302 (1.236, 1.371) | <.0001 | 1.204 (1.112, 1.303) | <.0001 |
| **Race** |  |  |  |  |
| White | Ref. |  | Ref. |  |
| Black | 1.486 (1.407, 1.570) | <.0001 | 1.241 (1.144, 1.347) | <.0001 |
| Asian | 0.676 (0.444, 1.028) | 0.0672 | 0.808 (0.477, 1.369) | 0.4285 |
| Other/Unknown | 0.275 (0.215, 0.350) | <.0001 | 0.613 (0.412, 0.911) | 0.0154 |
| **Ethnicity** |  |  |  |  |
| Not Hispanic or Latino | Ref. |  | Ref. |  |
| Hispanic or Latino | 0.975 (0.807, 1.178) | 0.792 | 0.942 (0.721, 1.230) | 0.66 |
| Unknown | 0.702 (0.643, 0.766) | <.0001 | 0.950 (0.807, 1.119) | 0.5383 |
| Missing | 0.855 (0.714, 1.025) | 0.09 | 0.790 (0.563, 1.110) | 0.174 |
| **Residence** |  |  |  |  |
| Rural | Ref. |  | Ref. |  |
| Urban | 0.977 (0.915, 1.044) | 0.4887 | 1.018 (0.924, 1.122) | 0.7122 |
| **Hypertension** |  |  |  |  |
| No | Ref. |  | Ref. |  |
| Yes | 1.092 (1.027, 1.162) | 0.0051 | 0.781 (0.713, 0.855) | <.0001 |
| **Diabetes** |  |  |  |  |
| No | Ref. |  | Ref. |  |
| Yes | 1.526 (1.427, 1.632) | <.0001 | 1.313 (1.191, 1.447) | <.0001 |
| **AKI** |  |  |  |  |
| No | Ref. |  | Ref. |  |
| Yes | 23.624 (22.321, 25.002) | <.0001 | 6.192 (5.671, 6.761) | <.0001 |

Table S4.1 Baseline characteristics among the subset with only one documented CoV-2 positive test.

| **Characteristics** | **Overall** | **No CKD** | **CKD** | **p-value** |
| --- | --- | --- | --- | --- |
|  | **N=666,778** | **N=658,866** | **N=7,912** |  |
| **Age group** |  |  |  | <.0001 |
| 18-29 | 171,649 (25.74) | 171,568 (26.04) | 81 (1.02) |  |
| 30-39 | 127,463 (19.12) | 127,277 (19.32) | 186 (2.35) |  |
| 40-49 | 114,792 (17.22) | 114,335 (17.35) | 457 (5.78) |  |
| 50-59 | 108,710 (16.30) | 107,703 (16.35) | 1,007 (12.73) |  |
| 60+ | 144,164 (21.62) | 137,983 (20.94) | 6,181 (78.12) |  |
| **Gender** |  |  |  | <.0001 |
| Female | 366,145 (54.91) | 362,532 (55.02) | 3,613 (45.66) |  |
| Male | 300,633 (45.09) | 296,334 (44.98) | 4,299 (54.34) |  |
| **Race** |  |  |  | <.0001 |
| White | 403,553 (60.52) | 399,012 (60.56) | 4,541 (57.39) |  |
| Black | 164,117 (24.61) | 160,882 (24.42) | 3,235 (40.89) |  |
| Asian | 6,915 (1.04) | 6,880 (1.04) | 35 (0.44) |  |
| Other/Unknown | 92,193 (13.83) | 92,092 (13.98) | 101 (1.28) |  |
| **Ethnicity** |  |  |  | <.0001 |
| Not Hispanic or Latino | 478,552 (71.77) | 471,687 (71.59) | 6,865 (86.77) |  |
| Hispanic or Latino | 39,927 (5.99) | 39,752 (6.03) | 175 (2.21) |  |
| Unknown | 123,543 (18.53) | 122,826 (18.64) | 717 (9.06) |  |
| Missing | 24,756 (3.71) | 24,601 (3.73) | 155 (1.96) |  |
| **Residence** |  |  |  | <.0001 |
| Rural | 95,495 (14.32) | 93,933 (14.26) | 1,562 (19.74) |  |
| Urban | 571,283 (85.68) | 564,933 (85.74) | 6,350 (80.26) |  |
| **Hypertension** |  |  |  | <.0001 |
| No | 574,513 (86.16) | 569,985 (86.51) | 4,528 (57.23) |  |
| Yes | 92,265 (13.84) | 88,881 (13.49) | 3,384 (42.77) |  |
| **Diabetes** |  |  |  | <.0001 |
| No | 626,624 (93.98) | 620,969 (94.25) | 5,655 (71.47) |  |
| Yes | 40,154 (6.02) | 37,897 (5.75) | 2,257 (28.53) |  |
| **AKI** |  |  |  | <.0001 |
| No | 649,703 (97.44) | 646,309 (98.09) | 3,394 (42.90) |  |
| Yes | 17,075 (2.56) | 12,557 (1.91) | 4,518 (57.10) |  |
| **Symptom** |  |  |  | <.0001 |
| Asymptomatic | 389,401 (58.40) | 385,116 (58.45) | 4,285 (54.16) |  |
| Mild | 204,814 (30.72) | 203,097 (30.83) | 1,717 (21.70) |  |
| Moderate/Severe | 72,563 (10.88) | 70,653 (10.72) | 1,910 (24.14) |  |
| **Hospitalization** |  |  |  | <.0001 |
| No | 646,106 (96.90) | 640,673 (97.24) | 5,433 (68.67) |  |
| Yes | 20,672 (3.10) | 18,193 (2.76) | 2,479 (31.33) |  |
| **ICU** |  |  |  | <.0001 |
| No | 663,196 (99.46) | 655,852 (99.54) | 7,344 (92.82) |  |
| Yes | 3,582 (0.54) | 3,014 (0.46) | 568 (7.18) |  |
| **Respiratory support** |  |  |  | <.0001 |
| No | 655,272 (98.27) | 648,586 (98.44) | 6,686 (84.50) |  |
| Yes | 11,506 (1.73) | 10,280 (1.56) | 1,226 (15.50) |  |
| **Follow-up time (days) [Median, min, max)** | 355, 1, 680 | 356, 1, 680 | 22, 1, 622 |  |

Table S4.2 Cox Proportional Hazard model for subsequent chronic kidney disease among subset of people with only one documented CoV-2 infection.

| **Characteristics** | **HR (95% C.I.)** | **p-value** |
| --- | --- | --- |
| **Age group** |  |  |
| 18-29 | Ref. |  |
| 30-39 | 2.799 (2.156, 3.632) | <.0001 |
| 40-49 | 6.484 (5.119, 8.212) | <.0001 |
| 50-59 | 11.657 (9.290, 14.628) | <.0001 |
| 60+ | 33.758 (27.058, 42.117) | <.0001 |
| **Gender** |  |  |
| Female | Ref. |  |
| Male | 1.314 (1.256, 1.374) | <.0001 |
| **Race** |  |  |
| White | Ref. |  |
| Black | 1.455 (1.388, 1.524) | <.0001 |
| Asian | 0.738 (0.529, 1.029) | 0.073 |
| Other/Unknown | 0.300 (0.244, 0.368) | <.0001 |
| **Ethnicity** |  |  |
| Not Hispanic or Latino | Ref. |  |
| Hispanic or Latino | 1.054 (0.903, 1.230) | 0.5076 |
| Unknown | 0.711 (0.658, 0.769) | <.0001 |
| Missing | 0.817 (0.696, 0.959) | 0.0134 |
| **Residence** |  |  |
| Rural | Ref. |  |
| Urban | 0.978 (0.924, 1.034) | 0.4263 |
| **Hypertension** |  |  |
| No | Ref. |  |
| Yes | 0.998 (0.947, 1.052) | 0.9395 |
| **Diabetes** |  |  |
| No | Ref. |  |
| Yes | 1.487 (1.405, 1.575) | <.0001 |
| **AKI** |  |  |
| No | Ref. |  |
| Yes | 21.104 (20.079, 22.181) | <.0001 |
| **ICU** |  |  |
| No | Ref. |  |
| Yes | 1.752 (1.604, 1.913) | <.0001 |

Table S5.1: Demographic and clinical characteristics for CoV-2 positive individuals with later end stage renal disease (N18.6) diagnosis.

| **Characteristics** | **Overall** | **No** | **Yes** | **p-value** |
| --- | --- | --- | --- | --- |
|  | **N=683,958** | **N=683,506** | **N=452** |  |
| **Age group** |  |  |  | <.0001 |
| 18-29 | 176,626 (25.82) | 176,615 (25.84) | 11 (2.43) |  |
| 30-39 | 130,568 (19.09) | 130,538 (19.10) | 30 (6.64) |  |
| 40-49 | 117,483 (17.18) | 117,425 (17.18) | 58 (12.83) |  |
| 50-59 | 111,164 (16.25) | 111,066 (16.25) | 98 (21.68) |  |
| 60+ | 148,117 (21.66) | 147,862 (21.63) | 255 (56.42) |  |
| **Gender** |  |  |  | <.0001 |
| Female | 376,331 (55.02) | 376,148 (55.03) | 183 (40.49) |  |
| Male | 307,627 (44.98) | 307,358 (44.97) | 269 (59.51) |  |
| **Race** |  |  |  | <.0001 |
| White | 414,156 (60.55) | 414,006 (60.57) | 150 (33.19) |  |
| Black | 169,297 (24.75) | 169,011 (24.73) | 286 (63.27) |  |
| Asian | 7,104 (1.04) | * | * |  |
| Other/Unknown | 93,401 (13.66) | * | * |  |
| **Ethnicity** |  |  |  | <.0001 |
| Not Hispanic or Latino | 492,632 (72.03) | 492,248 (72.02) | 384 (84.96) |  |
| Hispanic or Latino | 40,927 (5.98) | 40,903 (5.98) | 24 (5.31) |  |
| Unknown | 125,377 (18.33) | * | * |  |
| Missing | 25,022 (3.66) | * | * |  |
| **Residence** |  |  |  | <.0001 |
| Rural | 98,071 (14.34) | 97,967 (14.33) | 104 (23.01) |  |
| Urban | 585,887 (85.66) | 585,539 (85.67) | 348 (76.99) |  |
| **Hypertension** |  |  |  | <.0001 |
| No | 588,729 (86.08) | 588,395 (86.08) | 334 (73.89) |  |
| Yes | 95,229 (13.92) | 95,111 (13.92) | 118 (26.11) |  |
| **Diabetes** |  |  |  | <.0001 |
| No | 642,428 (93.93) | 642,057 (93.94) | 371 (82.08) |  |
| Yes | 41,530 (6.07) | 41,449 (6.06) | 81 (17.92) |  |
| **AKI** |  |  |  | <.0001 |
| No | 665,986 (97.37) | 665,789 (97.41) | 197 (43.58) |  |
| Yes | 17,972 (2.63) | 17,717 (2.59) | 255 (56.42) |  |
| **Symptom** |  |  |  | <.0001 |
| Asymptomatic | 399,921 (58.47) | 399,669 (58.47) | 252 (55.75) |  |
| Mild | 209,543 (30.64) | 209,446 (30.64) | 97 (21.46) |  |
| Moderate/Severe | 74,494 (10.89) | 74,391 (10.88) | 103 (22.79) |  |
| **Hospitalization** |  |  |  | <.0001 |
| No | 662,640 (96.88) | 662,330 (96.90) | 310 (68.58) |  |
| Yes | 21,318 (3.12) | 21,176 (3.10) | 142 (31.42) |  |
| **ICU** |  |  |  | <.0001** |
| No | 680,300 (99.47) | 679,896 (99.47) | 404 (89.38) |  |
| Yes | 3,658 (0.53) | 3,610 (0.53) | 48 (10.62) |  |
| **Respiratory support** |  |  |  | <.0001 |
| No | 672,115 (98.27) | 671,737 (98.28) | 378 (83.63) |  |
| Yes | 11,843 (1.73) | 11,769 (1.72) | 74 (16.37) |  |

Note: * small number less than 10 were masked due to DHEC’s policy.

** P-value from Fisher’s exact test.

Table S5.2: Demographic and clinical characteristics for CoV-2 positive individuals with later end stage renal disease (Z99.2) diagnosis.

| **Characteristics** | **Overall** | **No** | **Yes** | **p-value** |
| --- | --- | --- | --- | --- |
|  | **N=683,958** | **N=683,679** | **N=279** |  |
| **Age group** |  |  |  | <.0001 |
| 18-29 | 176,626 (25.82) | * | * |  |
| 30-39 | 130,568 (19.09) | * | * |  |
| 40-49 | 117,483 (17.18) | 117,442 (17.18) | 41 (14.70) |  |
| 50-59 | 111,164 (16.25) | 111,110 (16.25) | 54 (19.35) |  |
| 60+ | 148,117 (21.66) | 147,956 (21.64) | 161 (57.71) |  |
| **Gender** |  |  |  | <.0001 |
| Female | 376,331 (55.02) | 376,215 (55.03) | 116 (41.58) |  |
| Male | 307,627 (44.98) | 307,464 (44.97) | 163 (58.42) |  |
| **Race** |  |  |  | <.0001 |
| White | 414,156 (60.55) | 414,065 (60.56) | 91 (32.62) |  |
| Black | 169,297 (24.75) | 169,120 (24.74) | 177 (63.44) |  |
| Asian | 7,104 (1.04) | * | * |  |
| Other/Unknown | 93,401 (13.66) | * | * |  |
| **Ethnicity** |  |  |  | <.0001 |
| Not Hispanic or Latino | 492,632 (72.03) | 492,397 (72.02) | 235 (84.23) |  |
| Hispanic or Latino | 40,927 (5.98) | 40,908 (5.98) | 19 (6.81) |  |
| Unknown | 125,377 (18.33) | * | * |  |
| Missing | 25,022 (3.66) | * | * |  |
| **Residence** |  |  |  | <.0001 |
| Rural | 98,071 (14.34) | 98,007 (14.34) | 64 (22.94) |  |
| Urban | 585,887 (85.66) | 585,672 (85.66) | 215 (77.06) |  |
| **Hypertension** |  |  |  | <.0001 |
| No | 588,729 (86.08) | 588,529 (86.08) | 200 (71.68) |  |
| Yes | 95,229 (13.92) | 95,150 (13.92) | 79 (28.32) |  |
| **Diabetes** |  |  |  | <.0001 |
| No | 642,428 (93.93) | 642,202 (93.93) | 226 (81.00) |  |
| Yes | 41,530 (6.07) | 41,477 (6.07) | 53 (19.00) |  |
| **AKI** |  |  |  | <.0001 |
| No | 665,986 (97.37) | 665,854 (97.39) | 132 (47.31) |  |
| Yes | 17,972 (2.63) | 17,825 (2.61) | 147 (52.69) |  |
| **Symptom** |  |  |  | <.0001 |
| Asymptomatic | 399,921 (58.47) | 399,756 (58.47) | 165 (59.14) |  |
| Mild | 209,543 (30.64) | 209,484 (30.64) | 59 (21.15) |  |
| Moderate/Severe | 74,494 (10.89) | 74,439 (10.89) | 55 (19.71) |  |
| **Hospitalization** |  |  |  | <.0001 |
| No | 662,640 (96.88) | 662,436 (96.89) | 204 (73.12) |  |
| Yes | 21,318 (3.12) | 21,243 (3.11) | 75 (26.88) |  |
| **ICU** |  |  |  | <.0001** |
| No | 680,300 (99.47) | 680,043 (99.47) | 257 (92.11) |  |
| Yes | 3,658 (0.53) | 3,636 (0.53) | 22 (7.89) |  |
| **Respiratory support** |  |  |  | <.0001** |
| No | 672,115 (98.27) | 671,871 (98.27) | 244 (87.46) |  |
| Yes | 11,843 (1.73) | 11,808 (1.73) | 35 (12.54) |  |

Note: * small number less than 10 were masked due to DHEC’s policy.

** P-value from Fisher’s exact test.
